# Deconstructing Retinal Organoids: Single Cell RNA‐Seq Reveals the Cellular Components of Human Pluripotent Stem Cell‐Derived Retina

**DOI:** 10.1002/stem.2963

**Published:** 2019-01-12

**Authors:** Joseph Collin, Rachel Queen, Darin Zerti, Birthe Dorgau, Rafiqul Hussain, Jonathan Coxhead, Simon Cockell, Majlinda Lako

**Affiliations:** ^1^ Institute of Genetic Medicine Newcastle University Newcastle upon Tyne United Kingdom; ^2^ Bioinformatics Support Unit Newcastle University Newcastle upon Tyne United Kingdom; ^3^ Genomics Core Facility Newcastle University Newcastle upon Tyne United Kingdom

**Keywords:** Pluripotent stem cells, Single cell RNA‐Seq, Retinal organoids

## Abstract

The rapid improvements in single cell sequencing technologies and analyses afford greater scope for dissecting organoid cultures composed of multiple cell types and create an opportunity to interrogate these models to understand tissue biology, cellular behavior and interactions. To this end, retinal organoids generated from human embryonic stem cells (hESCs) were analyzed by single cell RNA‐sequencing (scRNA‐Seq) at three time points of differentiation. Combinatorial data from all time points revealed the presence of nine clusters, five of which corresponded to key retinal cell types: retinal pigment epithelium (RPE), retinal ganglion cells (RGCs), cone and rod photoreceptors, and Müller glia. The remaining four clusters expressed genes typical of mitotic cells, extracellular matrix components and those involved in homeostasis. The cell clustering analysis revealed the decreasing presence of mitotic cells and RGCs, formation of a distinct RPE cluster, the emergence of cone and rod photoreceptors from photoreceptor precursors, and an increasing number of Müller glia cells over time. Pseudo‐time analysis resembled the order of cell birth during retinal development, with the mitotic cluster commencing the trajectory and the large majority of Müller glia completing the time line. Together, these data demonstrate the feasibility and potential of scRNA‐Seq to dissect the inherent complexity of retinal organoids and the orderly birth of key retinal cell types. Stem Cells *2019;37:593–598*

**Significance Statement**

The rapid improvements in single cell sequencing technologies have opened new opportunities for dissecting the complexity of organoids derived from stem or primary cells. To demonstrate the feasibility of this approach, single cell RNA‐sequencing on retinal organoids was performed, which revealed the presence of multiple retinal cell types and their sequential emergence during the differentiation time course. Data show that this method has great potential for identifying multiple cell types arising within complex organoids, enabling detailed molecular and temporal systematic studies and close comparisons between in vitro derived tissues and in vivo organogenesis.

## Introduction

It has been estimated that 285 million people are affected by visual impairment globally, with retinal diseases accounting for approximately 26% of blindness 1. Many inherited and age‐related retinal dystrophies culminate in the loss of photoreceptors 2, 3. There are currently no treatments to reverse this degeneration, thus cell replacement has become a prerequisite on the path toward therapeutic transplantations. The derivation of human embryonic stem cells (hESCs) in 1998 4 and induced pluripotent stem cells (hiPSCs) in 2007 5 has provided the much needed breakthrough as both cell types can be expanded indefinitely in vitro as well as being able to generate photoreceptors and retinal pigment epithelium (RPE) 2, 3. The subsequent development of protocols to derive three‐dimensional retinal organoids from hESCs/hiPSCs demonstrated that structures akin to the developing eye and laminated retina arise and many retinal cell types are produced 6, 7. Despite these rapid advancements, methods to accurately define and characterize the cells that arise within these organoids over time have not been fully realized.

The improvements in next generation sequencing technologies and protocols for the application of these at a single cell level have broadened their application to multiple systems 8, 9. A few pioneering studies have applied single cell RNA sequencing (scRNA‐Seq) in human retinal tissue and organoids 10, 11, 12. These have largely focused on enriched specific cell types through the use of reporters or bait genes. Despite these important studies, we postulated that the full complexity of retinal organoids is yet to be fully resolved by scRNA‐Seq. In this study we used high throughput Integrated Fluidic Circuits (IFC) for scRNA‐Seq with a capacity of 800 capture sites to interrogate hESC‐derived retinal organoids through a differentiation time course. Using a greater cell capture number and an unselected single cell population allowed us to perform cell typing of the organoid composition and to follow the development of cell type emergence through the differentiation.

## Materials and Methods

A detailed description of all experimental procedures is presented in the Supporting Information.

## Results and Discussion

**Figure 1:**
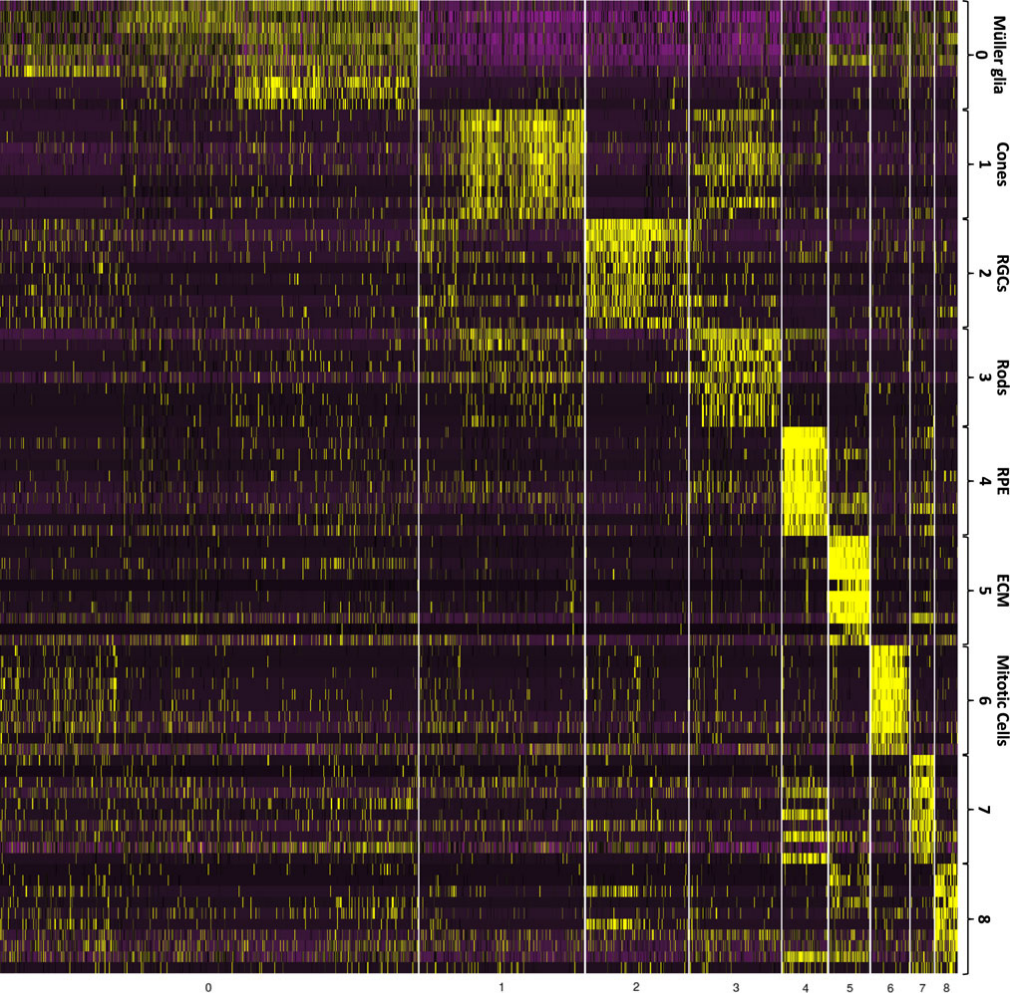
Clustering analysis reveals the presence of nine cell clusters. Seurat was used to align all time points to generate a combined data set. Clusters were then found and marker genes for each cluster identified and used to annotate them. The top 10 markers used for cluster annotation are shown in Supporting Information Table S1.

A hESC (H9) cell line was differentiated to retinal organoids. Samples were collected at 60, 90, and 200 days, dissociated, partitioned into single cells using the Fluidigm C1 Single‐Cell mRNA‐Seq HT IFC and processed for scRNA‐Seq. Following quality control and filtering (Supporting Information Fig. S1), data from scRNA‐Seq of each time point were normalized (Supporting Information Fig. S2) and then merged using the Seurat package to allow analysis of a higher cell number (1,976 single cells). The findCluster function revealed nine distinct clusters (Fig. 1; Supporting Information Table S1 and Fig. S3). The findMarkers function found the marker genes for each cluster 13. The top 10 marker genes were used to manually identify each cluster. Five of the clusters could be assigned to a certain cell type, with cluster 0 identified with genes commonly expressed in Müller glia, cluster 1 to cone photoreceptors, cluster 2 to retinal ganglion cells (RGCs), cluster 3 to rod photoreceptors and cluster 4 to RPE cells (Fig. 1). Cluster 5 expressed a number of extracellular matrix (ECM) genes shown to be expressed in retinal cells 14, 15, whereas cluster 6 expressed genes associated with mitosis, indicating a progenitor population. The remaining minor clusters 7 and 8 were comprised largely of ECM components, signaling molecules and metabolites, the majority of which have been proposed to play a role in retinal homeostasis (for example 16, 17, 18, 19).

**Figure 2:**
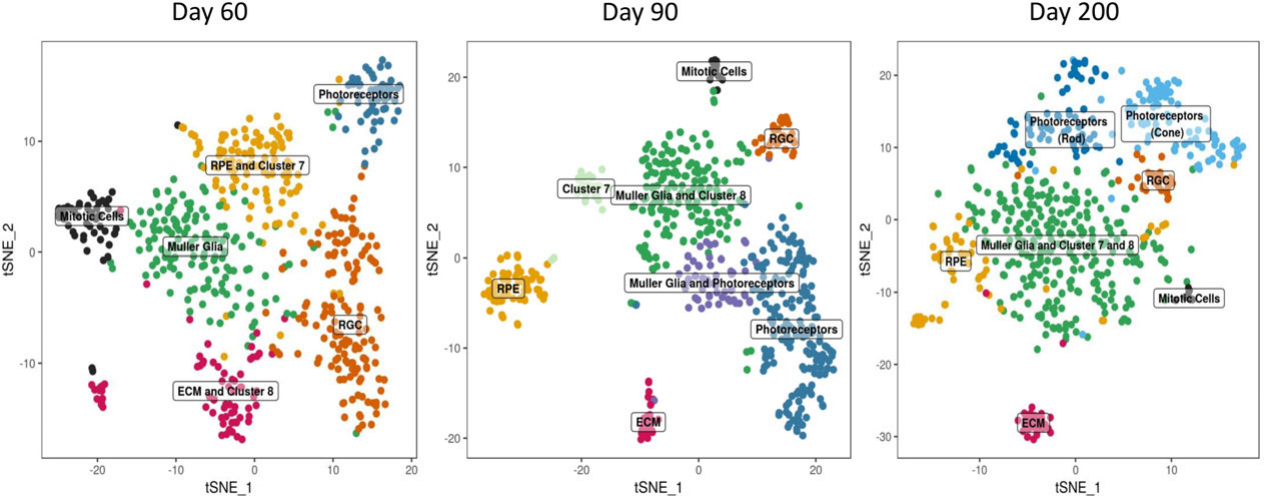
Comparison of individual clustering analysis at days 60, 90, and 200 of differentiation. Clusters for each time point were generated using Seurat and annotated using known retinal marker genes. The cells at different time points for each individual data set were down sampled to an equal number of 578 to ensure that the number of cells did not affect the number of clusters generated.

**Figure 3:**
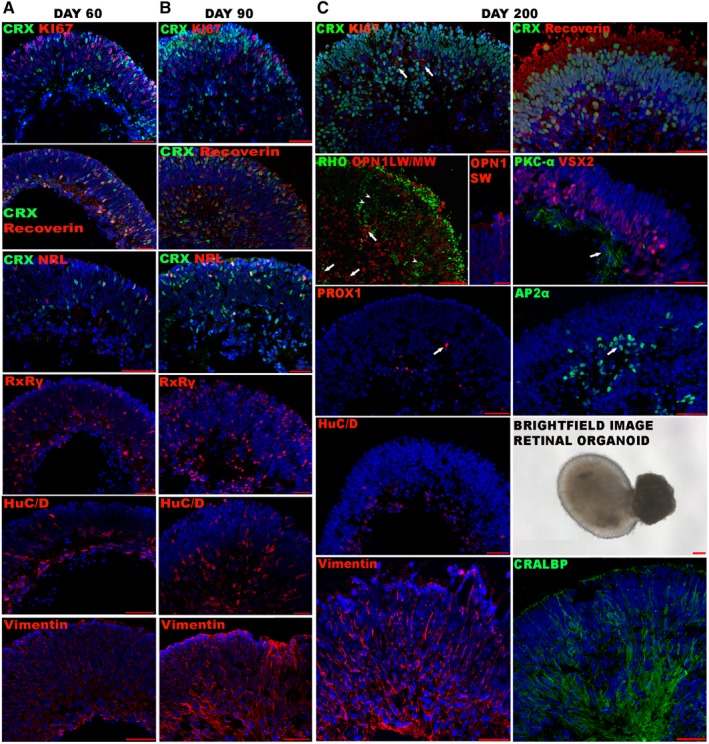
Immunohistochemical analysis of retinal organoids through the differentiation time course. Sections through retinal organoids at day 60 **(A)** and day 90 **(B)** using antibodies against: KI67 (red), CRX (green), Recoverin (red), NRL (red), RXRγ (red), HuC/D (red), and Vimentin (red) along with the nuclear stain Hoechst 33342 (blue). **(C):** Sections through retinal organoids at day 200 showing expression of selected retinal markers using antibodies against CRX (green), KI67 (red), Recoverin (red), rhodopsin (RHO; green, white arrowhead), OPN1LW/MW (red, white arrow), OPN1SW (red), PKC‐α (green, white arrow), VSX2 (red), PROX1 (red, white arrow), AP2α (green, white arrow), HuC/D (red), brightfield image of a retinal organoid, Vimentin (red), and CRALBP (green). Scale bars = 50 μm except OPN1SW scale bar = 20 μm and brightfield image retina organoid at day 200 scale bar = 100 μm.

Furthermore, clustering analyses were then performed on each time point (Fig. 2). This was compared with the combined data by superimposing the original clusters (identified in Fig. 1 and Supporting Information Fig. S3) to those identified at each individual time point (Fig. 2). This analysis revealed changes over the timeline of differentiation. For example, mitotic cells and RGCs decreased over time, photoreceptors resolved into cones and rods, a distinct RPE cluster formed and Müller glia cells increased toward day 200, recapitulating retinal development where Müller glia are the last retinal cell type to become postmitotic 20, 21, 22. To confirm this increase in complexity the percentage of the cell types at each time point were compared. Mitotic cells decreased from 7.7% at day 60 to 2.4% at day 200, RGCs decreased from 21% at day 60 to 6.6% at 200 and Müller glia increased from 43.7% at day 60 to 53% at day 200. This was corroborated by immunohistochemical analysis, which indicated the decreased presence of mitotic cells and RGCs from day 60 to day 200, stepwise photoreceptor differentiation (shown by the presence of Recoverin photoreceptor precursors, NRL rod precursors and RxRy cone precursors at days 60 and 90 and separation of rod and cone photoreceptors at day 200) and increased Müller glia presence from day 60 to 200, shown by Vimentin immunostaining from day 60 to 200 and the expression of CRALBP at day 200 (Fig. 3A, 3C). It is interesting to note that although horizontal (PROX1), rod bipolar (PKC‐α), and amacrine (AP2α)‐like cells were detected by immunohistochemical staining these were not depicted by scRNA‐Seq analysis. Therefore, to be able to resolve such clusters it may be necessary to increase the cell numbers analyzed. Although the decreased presence of mitotic cells during the differentiation time course was expected, that of RGCs was not and could be due to either culture conditions, which are not able to maintain this cell type for prolonged periods, or programmed cell death as seen during development 20, 23.

Interestingly, this analysis also depicted cell types with transcriptional profiles shared by several clusters (Fig. 2). For example, at day 60, RPE shared transcriptional similarity with cluster 7 and ECM with cluster 8. At day 90, cell clusters with transcriptional profiles shared by Müller glia and photoreceptors, corroborating findings reported in adult murine retina 24 but not transcriptionally documented in human retinal development as yet, and Müller glia and cluster 8 were identified. Over time, the shared transcriptome diminished and distinct clusters emerged, with the exception of Müller glia cells, which retained a shared transcriptional profile with clusters 7 and 8.

**Figure 4:**
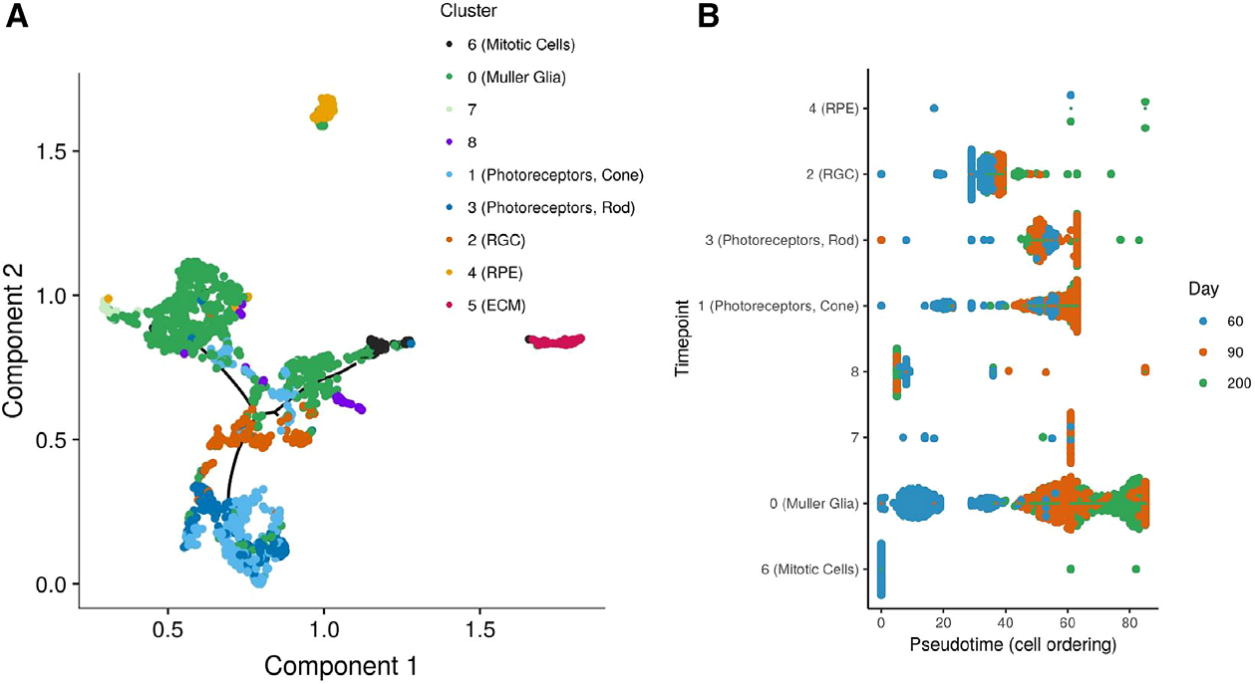
Pseudo‐time analysis reveals the emergence of various retinal cell types during the differentiation process. **(A):** A pseudo‐time trajectory from the RPE, RGC, Müller glia, cone, and rod photoreceptor clusters was constructed using monocle. **(B):** Order of cell emergence is shown by day and cell type.

As the complexity of the organoids increased and distinct cell types could be resolved over time additional pseudo‐time analysis was conducted. Monocle 25 was used to analyze the highly variable genes from the mitotic, RPE, RGC, Müller glia, cone, and the rod clusters (Fig. 4A)**.** This analysis identified the mitotic cluster as the proliferating population from which the rest of the cells emerged. A Müller glia subpopulation resides next along this branch. This is potentially due to the expression of genes commonly expressed in retinal progenitor cells (RPCs) within the Müller glia cluster (Supporting Information Fig. S4), corroborating published data for murine Müller glia cells 24, 26. Continuing down this branch, a few cone‐like photoreceptors begin to arise, here the pseudo‐time trajectory forked with an upper branch bearing the majority and the remaining Müller glia cells along with minor cluster 7, which was characterized by genes that have been shown to be involved in retinal homeostasis, in which Müller glia play an important role. The lower branch is initially populated with RGCs and then terminates with two closely related clusters containing the rod and cone photoreceptors. This pseudo‐time trajectory resembles the order of retinal cell development with a progenitor population giving rise firstly to RGCs, followed by cones, then rods and finally the majority of Müller glia, which are situated on a separate branch, potentially indicating their unique role in retinal development and homeostasis, and thus their transcriptome. The RPE and ECM clusters reside outside of this trajectory. RPE are known to differentiate relatively early in development during optic cup formation 27 and thus have a more distinct transcriptome. The genes from the ECM cluster are not exclusive to the retina, being common ECM components expressed throughout many tissue types and thus by ontology are unlikely to be associated with the retina. This is likely to explain why these two clusters are not associated with the time line (Fig. 4A). The cell ordering plot (Fig. 4B) further corroborates the order of cell type emergence within the organoids with mitotic cells, some Müller glia and cluster 8 present initially at the first time point. Cone photoreceptors and RGCs are next, arising at day 60, followed with diminishing RGC birth and the majority of cones appearing on day 90, alongside the emergence of rods and more Müller glia. A large number of Müller glia cells (along with some rods) were present at day 200 as the later cell types to mature.

## Conclusion

Our data demonstrate the feasibility and potential of scRNA‐Seq to dissect the inherent complexity of retinal organoids and the orderly birth of key retinal cell types therein, which recapitulates the order of retinal development.

## Author Contributions

J. Collin: study design, performed research, data collection and analysis, figure preparation, manuscript writing; R.Q.: data analysis, figure preparation, manuscript writing; D.Z., B.D.: performed research, data collection and analysis, contributed to manuscript writing; R.H., J. Coxhead: performed research, data collection; S.C.: data analysis; M.L.: study design, data analysis, figure preparation, manuscript writing, fund raising; J. Collin, R.Q., D.Z., B.D., R.H., J. Coxhead, S.C., and M.L.: approved the final version of the manuscript.

## Disclosure of Potential Conflicts of Interest

All authors indicated no potential conflicts of interest.

## Supporting information


**Appendix S1**: Supporting Information

**Figure S1 Stepwise filtering strategy of single cell RNA‐Seq data.** Quality control of the raw data was performed using the Scater R package and applied to each time point. (**A & B**) For day 60 the threshold was set to remove cells with fewer than 100,000 reads or 2000 genes (B). For day 90 and 200 a filter was applied to remove cells with fewer than 150,000 reads or 2000 genes (A); (**C**) Cells with higher than 15% of mitochondrial genes were removed; (**D**) For day 60 and 200 cells containing higher than 15% of Ambion spikes were removed from the analysis. For day 90 this threshold was set at 75%. (**E**) The range of total reads per cell aligned to endogenous genes.

**Figure S2 Contribution of known technical factors to variation between each dataset before and after normalization**. The Seurat CCA method was used to combine data from each time point. t‐SNE plots show the clustering of the cells before and after normalization.

**Figure S3 Combined t‐SNE plot of clustering analysis reveals the presence of nine cell clusters.** Seurat was used to align all time points to generate a combined dataset.

**Figure S4 Expression of retinal progenitor cell marker genes in the clusters present at each time point**. RPC marker genes *LIN28, ZIC1, DLL3, LGR5, SOX9, GLI1, VSX2, SFRP2, ASC1, SOX2, LHX2, PRTG, RAX, FGF19, HES1, PAX6, SIX3* and *SIX6* were used to assess expression in the clusters over time. A combined violin plot shows the total expression of these genes within the clusters. A Wilcoxon test was used to compare total expression of the RPC genes between the clusters. Both the mitotic and the Müller glia cluster showed significant expression of the RPC genes with p values of 7.066140e‐07 and 2.724621e‐40 respectively at day 60. The Müller glia cluster showed significant expression of RPC genes at day 90 (p value = 7.298821e‐38) and day 200 (p value = 7.209557e‐52).

**Table S1** List of top ten markers used for cluster identification shown in Figure 1.

**Table S2** Summary of antibodies used for immunohistochemical staining.
