# Supplementary material for: Deconstructing Retinal Organoids: Single Cell RNA‐Seq Reveals the Cellular Components of Human Pluripotent Stem Cell‐Derived Retina
Source: Stem Cells. 2019 Jan 12;37(5):593–8. doi: 10.1002/stem.2963 (PMC6519347; doi:10.1002/stem.2963)
Supplement: Supplementary file 1 — Appendix S1: Supporting Information [file STEM-37-593-s001.docx]

**Materials and Methods**

*Human pluripotent stem cell culture and differentiation*

A human embryonic stem cell (hESC) line was expanded in mTeSR™1 (StemCell Technologies) at 37°C and 5% CO2 on 6 well plates pre-coated with Low Growth Factor Matrigel (Corning). Differentiation to retinal organoids was performed as described in Mellough et al. 2015 [1] with minor modifications, which included addition of 10 µM Y27632 dihydrochloride (Chemdea) for the first 48 hours of differentiation and 10% foetal calf serum, T3 (40 ng/ml; Sigma-Aldrich), Taurine (0.1 mM; Sigma-Aldrich) & Retinoic Acid (0.5 µM; Sigma-Aldrich) from day 18 of differentiation.

*Generation of single cell cDNA library for mRNA sequencing*

Retinal organoids were dissociated to single cells using the Embryoid Body Dissociation Kit (Miltenyi Biotech) following the manufacturer’s instructions. Single cells were loaded onto the C1 Single-Cell mRNA-Seq HT IFC [10-17 µm] (Fluidigm). Array Control RNA Spikes (Thermo Fisher Scientific) were prepared and added to lysis mix as described in the Fluidigm protocol. For our first experiment, conducted with samples obtained from differentiation day 90, Array Control RNA Spikes were added to the experiment following concentrations recommended by Fluidigm. A quality control check measuring the percentage spike in to endogenous gene revealed that this concentration was very high for our cell type and the concentration of spike in was diluted by 1:10 in subsequent experiments. Cell lysis, reverse transcription and cDNA amplification were performed using the SMART-Seq v4 Ultra Low Input RNA Kit for the Fluidigm C1 System (Clontech). Full length cDNA libraries were prepared using the Illumina Nextera XT DNA library preparation kit (Illumina). Libraries were pooled and sequenced (2 x 75 bp) on the Ilumina NextSeq 500 using a Mid Output v2 kit.

*Read Alignment and Quantification*

The Fluidigm mRNASeqHT_demultiplex.pl was used to demulitplex the fastq files into single cell FASTQ files. FASTQ files were trimmed with Trimmomatic version 0.33 with the parameters: trailing = 20, minlength = 60 and end = “PE”. The human reference genome GRCh38.p7 version 25 from GenCode was concatenated with the Ambion spike sequences provided by Fluidigm to create a reference genome. “Comprehensive gene annotation” was used for annotation and STAR 2.4.0 was used for alignment. A STAR index was created using the reference genome and annotations used with read length set to 75. STAR default parameters were used for alignment. The SAM files produced by STAR were converted into BAM files using SAMtools1.3. Reads were quantified using HTSEQ 0.6.1 with these parameters -f bam, -r name, -a 4, -i gene_id, -m union.

*Quality Control: Filtering Cells and Genes*

The Scater R package was used for quality control and initial visualization of the raw data. The quality control steps were applied to each individual time point. A quality control threshold was applied to remove any cells where fewer than 150000 reads or 2000 genes for all experiments, except day 60 where a filter of 100000 reads or 2000 genes was applied. Cells below these thresholds were removed from downstream analysis (**Figure S1A**, **B**). High levels of mitochondrial genes have been shown to be an indication of dead or poor-quality cells, thus cells with higher than 15% of mitochondrial genes were filtered (**Figure S1C**). For all time points, except day 90, cells containing higher than 15% of Ambion spikes were also removed from the analysis. The average level of Ambion spikes for the day 90 experiment was very high and this threshold was set at 75% (**Figure S1D**). Genes were filtered from analysis if they were detected in fewer than 2 cells (after cell filtering). Despite the differences in spike in concentration the number of reads per cell observed aligning to endogenous genes at day 90 fell within the range observed at day 60 and day 200 (**Figure S1E**). After filtering stage, 578 cells from day 60, 661 cells from day 90 and 737 cells from day 200 and 24415 genes passed quality control. Data is deposited in the Gene Expression Omnibus (GEO) accession number GSE119893.

*Combining Time Points and Clustering Analysis*

To adjust for read depth we used the Seurat R package (version 2.3.0) was used to normalise and scale the individual experiments using the “LogNormalize” method. The datasets were then merged using the Seurat alignment method, to overcome experiment specific variation, such as read depth and coverage. This method identifies shared sources of variation making it possible to detect stable cell populations across multiple experiments [2]. Firstly, the top 1000 variable genes were chosen for each dataset and Seurat canonical correlation analysis was performed to identify common sources of variation between the datasets.  This produced a combined dataset containing 1976 cells. Finally, the CCA subspaces were aligned. Figure S2 (top) shows the proportion of variance attributed to known technical factors such as read depth and experimental timepoint. We saw that these factors contributed to between 1-10% of the total variance before normalisation and this is then reduced to below 1% after normalisation. However as the tSNE plot in Figure S2 shows, despite normalisation there was a clear separation of cells by experimental condition when all three timepoints were combined. After the CCA alignment the cells clustered by biological type rather than day. The Seurat findCluster function, with a resolution of 0.6 was used to cluster cells, using highly variable genes [3]. The findMarkers function was used to identify markers for each cluster and the clusters were annotated using these genes.

*Analysis of Individual Time Points*

To compare the complexity of the cells at different time points each individual dataset was down-sampled to an equal number of cells (578 cells) so as not to affect the number of clusters generated. Clusters were then identified with findCluster, with a resolution of 0.8 within the individual datasets. These clusters were compared with the cell assignments from the previously annotated combined clustering analysis. Marker genes were again identified for the individual analysis.

*Pseudotime Analysis*

Monocle version 2.99 was used to construct a pseudotime trajectory. The down sampled data set consisting of 578 cells from each time point was used for pseudotime analysis. The highly variable genes identified by Seurat were used to create the trajectory. Once the trajectory was calculated, the Monocle differentialGeneTest was used to identify pseudotime dependent genes.

*Immunohistochemistry*

For immunohistochemistry (IHC) retinal organoids were collected on day 60, 90 and 200, fixed in 4% PFA for 20 minutes, followed by three washes in phosphate-buffered saline (PBS), incubated overnight in 30% sucrose/PBS, embedded in optimum cutting temperature (OCT) embedding matrix (Cellpath) and frozen at -20°C. 10 µm cryostat sections were collected using a Leica Cm1860 cryostat (Leica). Cryosections were air-dried, washed several times in PBS and incubated in blocking solution (10 % normal goat serum, 0.3 % Triton-X-100 in PBS) for one hour at room temperature. Slides were incubated with the appropriate primary antibody overnight 4°C (**Table S2**). After rinsing with PBS, sections were incubated with the secondary antibody for 2 hours at RT. Alexa Fluor 488 and 546 secondary antibodies (Invitrogen-Molecular Probes) were used at a 1:1000 dilution. Negative controls were carried out by omitting the primary antibody. Afterwards, sections were washed three times in PBS and mounted with Vectashield (Vector Laboratories, Burlingame, CA) containing 10 μg/ml Hoechst 33342 (Life Technologies, UK) for counterstaining nuclei.

*Image Acquisition and Analysis*

Bright‐field and fluorescent images were captured using an Axio Imager upright microscope with Apotome (Zeiss, Germany) structured illumination fluorescence using a 20x objective operated with AxioVision software. Final images are presented as a maximum projection and adjusted for brightness and contrast in Adobe Photoshop (Adobe Systems).

*References*

1 Mellough CB, Collin J, Khazim M, et al. IGF-1 Signaling Plays an Important Role in the Formation of Three-Dimensional Laminated Neural Retina and Other Ocular Structures from Human Embryonic Stem Cells. Stem Cells 2015;33:2416–2430.

2 Butler A, Hoffman P, Smibert P, et al. Integrating single-cell transcriptomic data across different conditions, technologies, and species. Nat Biotechnol 2018;36:411–420.

3 Brennecke P, Anders S, Kim JK, et al. Accounting for technical noise in single-cell RNA-seq experiments. Nat Methods 2013;10:1093–1095.
