# Supplementary figures and images for: Deconstructing Retinal Organoids: Single Cell RNA‐Seq Reveals the Cellular Components of Human Pluripotent Stem Cell‐Derived Retina
Source: Stem Cells. 2019 Jan 12;37(5):593–8. doi: 10.1002/stem.2963 (PMC6519347; doi:10.1002/stem.2963)

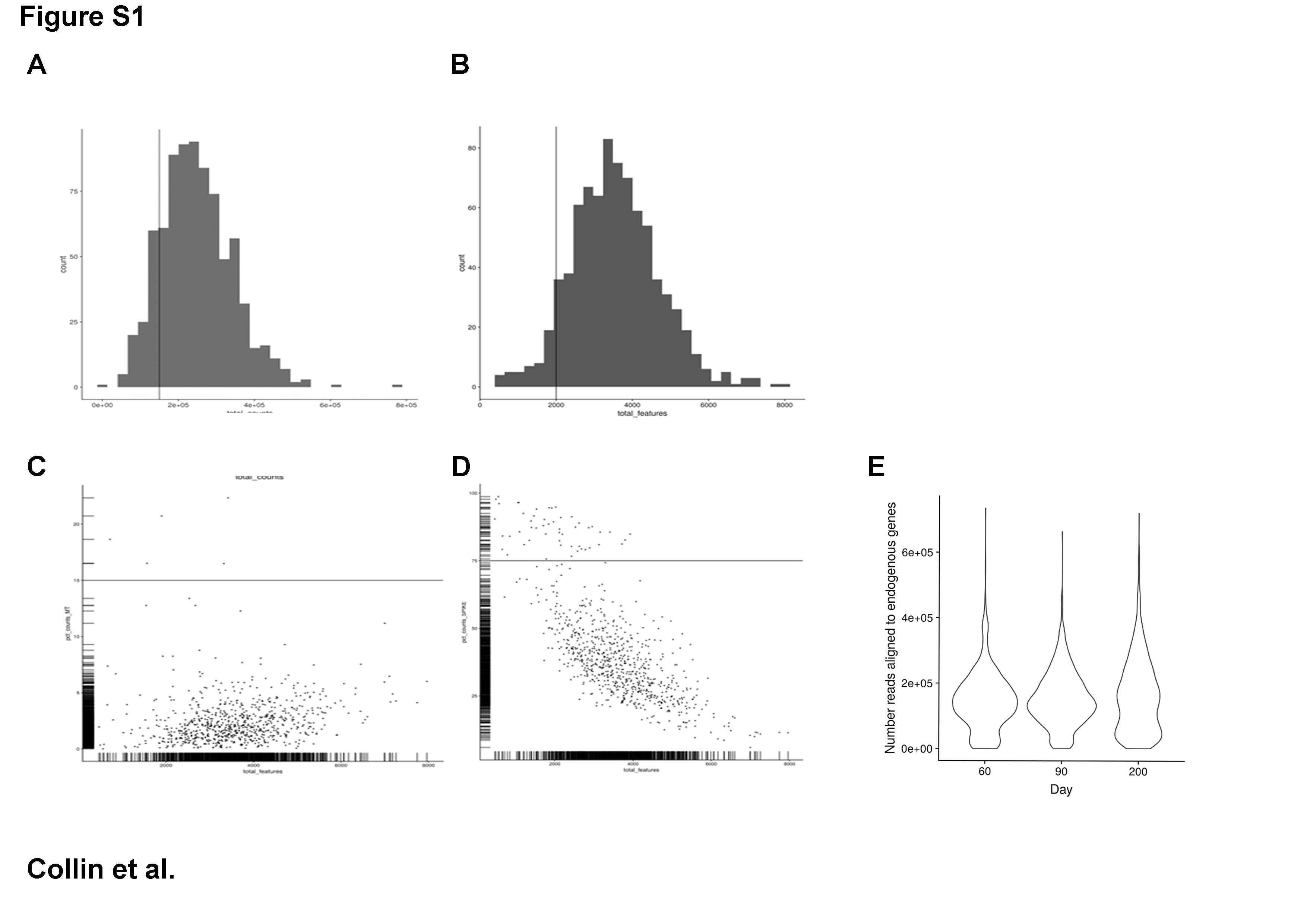

Supplement: Supplementary file 2 — Figure S1 Stepwise filtering strategy of single cell RNA‐Seq data. Quality control of the raw data was performed using the Scater R package and applied to each time point. (A & B) For day 60 the threshold was set to remove cells with fewer than 100,000 reads or 2000 genes (B). For day 90 and 200 a filter was applied to remove cells with fewer than 150,000 reads or 2000 genes (A); (C) Cells with higher than 15% of mitochondrial genes were removed; (D) For day 60 and 200 cells containing higher than 15% of Ambion spikes were removed from the analysis. For day 90 this threshold was set at 75%. (E) The range of total reads per cell aligned to endogenous genes. [file STEM-37-593-s002.jpg]

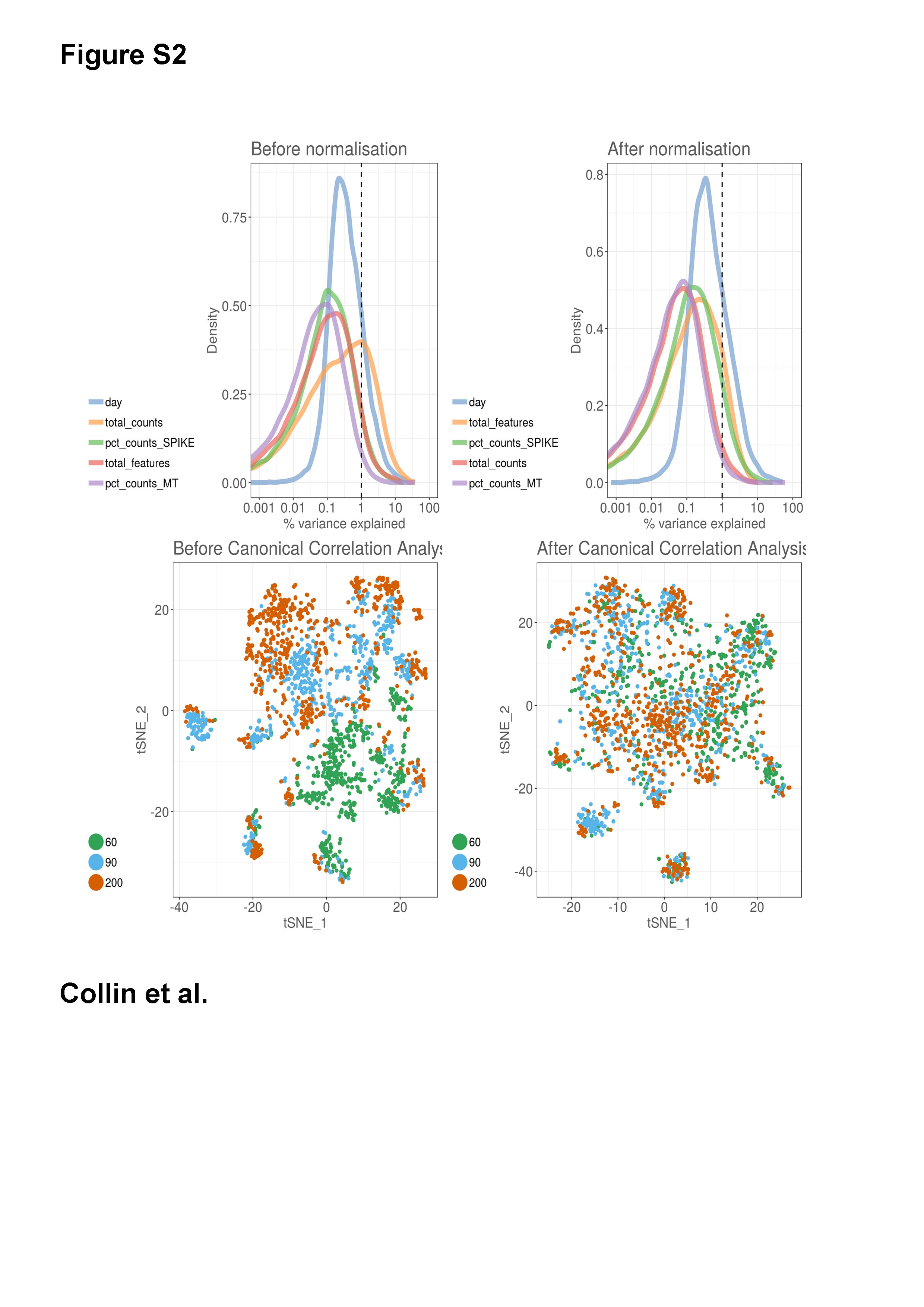

Supplement: Supplementary file 3 — Figure S2 Contribution of known technical factors to variation between each dataset before and after normalization. The Seurat CCA method was used to combine data from each time point. t‐SNE plots show the clustering of the cells before and after normalization. [file STEM-37-593-s003.jpg]

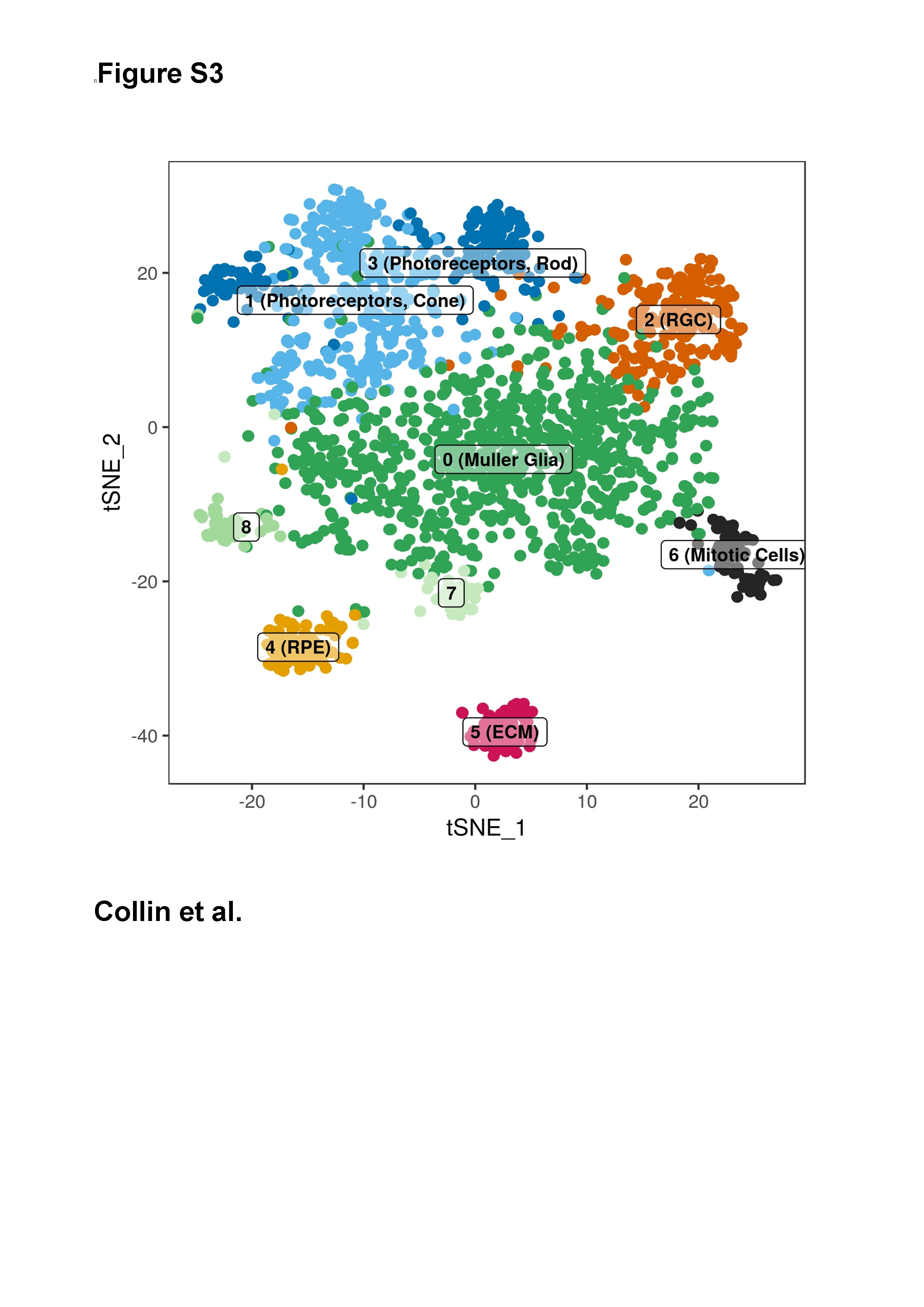

Supplement: Supplementary file 4 — Figure S3 Combined t‐SNE plot of clustering analysis reveals the presence of nine cell clusters. Seurat was used to align all time points to generate a combined dataset. [file STEM-37-593-s004.jpg]

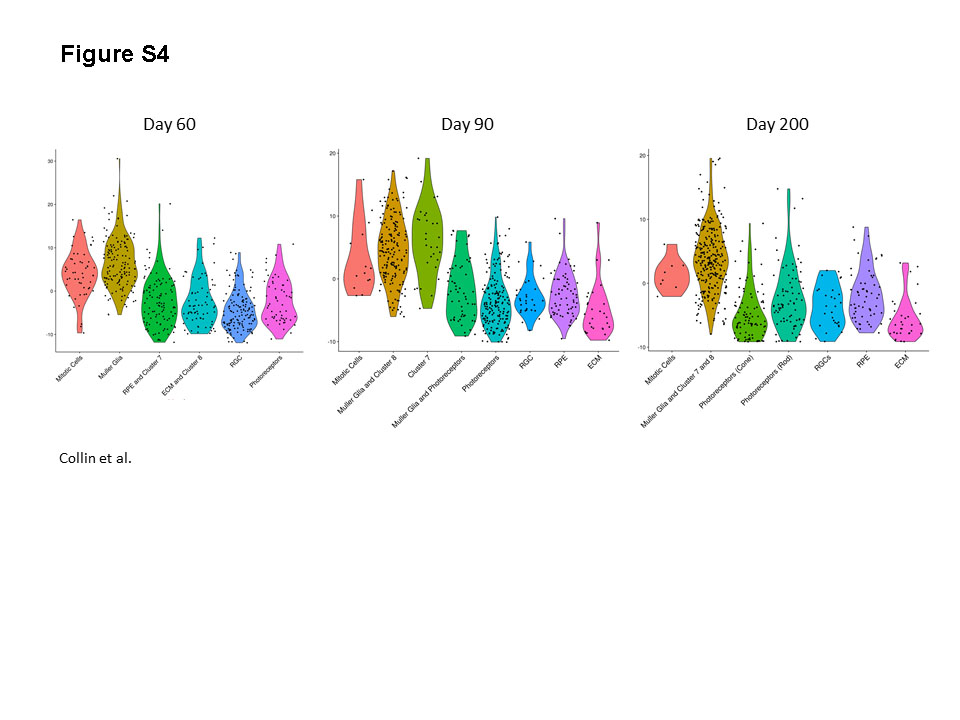

Supplement: Supplementary file 5 — Figure S4 Expression of retinal progenitor cell marker genes in the clusters present at each time point. RPC marker genes LIN28, ZIC1, DLL3, LGR5, SOX9, GLI1, VSX2, SFRP2, ASC1, SOX2, LHX2, PRTG, RAX, FGF19, HES1, PAX6, SIX3 and SIX6 were used to assess expression in the clusters over time. A combined violin plot shows the total expression of these genes within the clusters. A Wilcoxon test was used to compare total expression of the RPC genes between the clusters. Both the mitotic and the Müller glia cluster showed significant expression of the RPC genes with p values of 7.066140e‐07 and 2.724621e‐40 respectively at day 60. The Müller glia cluster showed significant expression of RPC genes at day 90 (p value = 7.298821e‐38) and day 200 (p value = 7.209557e‐52). [file STEM-37-593-s005.jpg]
