# Supplementary material for: Deconstructing Retinal Organoids: Single Cell RNA‐Seq Reveals the Cellular Components of Human Pluripotent Stem Cell‐Derived Retina
Source: Stem Cells. 2019 Jan 12;37(5):593–8. doi: 10.1002/stem.2963 (PMC6519347; doi:10.1002/stem.2963)
Supplement: Supplementary file 7 — Table S2 Summary of antibodies used for immunohistochemical staining. [file STEM-37-593-s007.docx]

| **Antibody** | **Host** | **Dilution** | **Supplier, Cat. No** |
| --- | --- | --- | --- |
| Anti-Crx | Mouse | 1:200 | Abnova, H00001406-M02 |
| Anti- Ki67 | Rabbit | 1:200 | Abcam, ab15580 |
| Anti-Nrl | Mouse | 1:200 | Santa Cruz, sc-374277 |
| Anti- CRALBP | Mouse | 1:100 | abcam, ab15051 |
| Anti-Ap2α | Mouse | 1:200 | Santa Cruz, sc-12726 |
| Anti-Recoverin | Rabbit | 1:1000 | Millipore, AB5585 |
| Anti-PKCα | Mouse | 1:200 | BD Transduction laboratories, 610107 |
| Anti-opsin Blue (OPN1SW) | Rabbit | 1:200 | Millipore, AB5407 |
| Anti-opsin Red/Green (OPN1LW/MW) | Rabbit | 1:200 | Millipore, AB5405 |
| Anti- Vimentin | Rabbit | 1:400 | gift from Prof. Roy Quinlan |
| Anti-HuC/D | Mouse | 1:200 | Invitrogen, A21271 |
| Anti-Prox1 | Rabbit | 1:1000 | Millipore, AB5475 |
| Anti-RetP1(Rhodopsin) | Mouse | 1:200 | Sigma, O4886 |
| Anti-RXRγ | Rabbit | 1:200 | Santa Cruz, sc555 |
| Anti-CHX10 | Rabbit | 1:50 | Sigma Atlas, HPA003436 |

**Table S2:** Summary of antibodies used for immunohistochemical staining.
